# A Game-Based School Program for Mental Health Literacy and Stigma on Depression (Moving Stories): Cluster Randomized Controlled Trial

**DOI:** 10.2196/26615

**Published:** 2022-08-17

**Authors:** Anouk Tuijnman, Marloes Kleinjan, Merlijn Olthof, Evert Hoogendoorn, Isabela Granic, Rutger CME Engels

**Affiliations:** 1 Behavioural Science Institute Radboud University Nijmegen Nijmegen Netherlands; 2 Trimbos-institute Utrecht Netherlands; 3 Department of Interdisciplinary Social Sciences Youth Studies Utrecht University Utrecht Netherlands; 4 IJsfontein Amsterdam Netherlands; 5 Faculty of Social Sciences McMaster University Hamilton, ON Canada; 6 Erasmus School of Social and Behavioural Sciences Erasmus University Rotterdam Netherlands

**Keywords:** depression, help-seeking behavior, helping behavior, health literacy, stigma, video games, adolescence, secondary schools, mental health, digital health

## Abstract

**Background:**

Depressive symptoms are highly prevalent among adolescents in Western countries. However, although treatment for depressive symptoms is available, many adolescents do not seek help when they need it. Important barriers to help-seeking among adolescents include low mental health literacy and high stigma. Therefore, we have developed a game-based school program, *Moving Stories*, which combines mental health literacy training for depression with contact with someone with lived experience both in the digital and nondigital world.

**Objective:**

The aim of this study is to conduct a first test of the effectiveness of the newly developed game-based program, *Moving Stories*, using a cluster randomized controlled trial.

**Methods:**

A total of 185 adolescents participated, divided over 10 classes from 4 schools. Half of the classes were randomly selected to follow the *Moving Stories* program, whereas the other half were in the control group, where no intervention was provided. The adolescents filled out digital questionnaires at 4 time points, with questions on mental health literacy, stigma, depressive symptoms, and the program itself (before the program, after the program, 3-month follow-up, and 6-month follow-up). Using R (R Foundation for Statistical Computing), we ran linear mixed-effects models for all continuous outcome variables and generalized linear mixed-effects models for all binary outcome variables.

**Results:**

Compared with the control group, participants in the *Moving Stories* group improved after the program in personal stigma (*b*=−0.53, 95% CI −1.02 to −0.03; t_179.16_=−2.08; *P*=.04). Effects on personal stigma lasted over time (3-month follow-up: *b*=−0.57, 95% CI −1.11 to −0.03; t_174.39_=−2.07; *P*=.04). Most adolescents in the *Moving Stories* group participated in the introduction (97/99, 98%) and contact session (93/99, 94%), played the game for 4 or 5 days (83/99, 83%), and indicated that they would recommend the game to their peers (90/98, 92%).

**Conclusions:**

The results of this study show the potential of *Moving Stories* as a stigma reduction program. With changes in the program to improve its effects on mental health literacy, *Moving Stories* could be implemented in schools to improve help-seeking in adolescents and reduce the negative consequences and burden of depressive symptoms.

**Trial Registration:**

Dutch Trial Register NTR7033; https://trialsearch.who.int/Trial2.aspx?TrialID=NTR7033

**International Registered Report Identifier (IRRID):**

RR2-10.2196/11255

## Introduction

### Background

Depressive symptoms are highly prevalent among adolescents in Western countries [1-3] and increase the risk of developing a depressive disorder later in life [4,5]. Both depressive symptoms and a depressive disorder have many negative consequences, including social, academic, and physical problems [6,7]. In extreme cases, a depressive disorder can lead to suicidal thoughts and suicide attempts [2,4]. Suicide is one of the main causes of death among youths worldwide [2]. Owing to these detrimental consequences, it is vital that adolescents seek help if they experience depressive symptoms. However, although treatment for depressive symptoms is available [8-12], many adolescents do not seek help when they need it [13-15]. Moreover, the longer it takes to receive help, the worse the response to treatment is [16].

Important barriers to help-seeking among adolescents are low mental health literacy and high stigma [13,17]. Mental health literacy comprises “knowledge and beliefs about mental disorders which aid their recognition, management or prevention” [18], whereas stigma “exists when elements of labeling, stereotyping, separation, status loss, and discrimination occur together in a power situation that allows them” [19]. Social support, on the other hand, increases help-seeking [13]. In light of the high prevalence and negative consequences of depressive symptoms, it is pivotal to lower barriers to help-seeking. Therefore, we developed a game-based school program, called *Moving Stories*, that targets mental health literacy and stigma on depression [20]. The aim of this study is to examine the effectiveness of this program.

### Mental Health Literacy and Stigma

Mental health literacy is a broad concept that not only concerns knowledge of mental health disorders but also refers to connected actions, both by those who need help themselves and by those close to them [18,21]. More specifically, Jorm [21] defined the main components of mental health literacy as “(a) knowledge of how to prevent mental disorders, (b) recognition of when a disorder is developing, (c) knowledge of help-seeking options and treatments available, (d) knowledge of effective self-help strategies for milder problems, and (e) first aid skills to support others who are developing a mental disorder or are in a mental health crisis.” The *Moving Stories* program targets recognition (component b), knowledge of help-seeking options and treatments (component c), and first aid skills (component e), specifically for depression in youth [20]. The goal of the *Moving Stories* program is to improve help-seeking. As component (a) of mental health literacy concerns the prevention of mental disorders and component (d) concerns knowledge of self-help strategies, we decided not to focus on these 2 components.

In adolescents, recognizing mental health disorders such as depression has been linked to choosing appropriate help [22] and increased help-seeking recommendations to peers in need [22,23]. However, most adolescents have trouble identifying depressive symptoms [24]. Adolescents also have limited knowledge of help-seeking options and available treatments. Often, they do not know how and where to seek help [25]. Moreover, adolescents prefer to seek help from people they know [23,26-28] and believe this help to be the most beneficial [29]. Unfortunately, seeking help solely from peers is inadvisable as peers are usually not able to provide the help that is warranted [21,30]. Therefore, improving symptom recognition, knowledge of appropriate help and treatment, and first aid skills in adolescents is important in furthering help-seeking.

Although improving symptom recognition is a good strategy to enhance help-seeking, recognizing depressive symptoms and labeling someone as mentally ill have also been linked to stigmatizing attitudes [31]. Both personal and perceived stigmatizing attitudes have been found to hinder help-seeking [13,32-35]. Personal stigma refers to a person’s “own attitudes to a mentally ill person,” whereas perceived stigma refers to a person’s “perceptions of the attitudes held by other people” [36]. High personal stigma in adolescents is also related to poorer first aid skills [37]. Therefore, programs that target mental health literacy should also, to a similar extent, focus on reducing stigmatizing attitudes.

### Stigma and Mental Health Literacy Programs

Several studies have tested the effectiveness of programs specifically aimed at reducing stigma toward mental health. Meta-analyses have shown that antistigma programs, in general, are effective in reducing stigma [38,39]. Successful elements of antistigma programs are education regarding mental health disorders and direct or indirect contact with someone who has experienced a mental health disorder. Research on mental health literacy programs among youth is scarce. Most programs that include components targeting mental health literacy primarily focus on enhancing mental health rather than literacy [40]. Moreover, most studies on mental health literacy lack a rigorous evaluation with a randomized design, validated measures, or follow-up measurements [40-42]. There are a few exceptions, and these more rigorous studies show promising results (eg, *Headstrong* [43] and *teen Mental Health First Aid* [44-47]). Despite these promising results, currently available mental health literacy programs for youth are subject to limitations. Moreover, there has been some criticism of the way contact sessions with someone with lived experience with a mental health disorder are organized in stigma programs for youth [48,49].

Few mental health literacy programs target behavioral mental health literacy components (ie, help-seeking and first aid behavior). Most programs aim to improve knowledge and attitudes but do not focus on changing behavior [21]. In addition, most mental health literacy programs are fairly didactic, whereas adolescents themselves prefer more interaction [40]. Studies on stigma programs in young adolescents have reported possible limitations of contact sessions, including that when effects are not found, it could be because of (1) the length of a contact session (eg, when it is just 1 hour [49]), (2) the way the person with lived experience is introduced (eg, when a surprise introduction is used, which distracts the adolescents from the provided information [48]), and (3) the age difference between the adolescents and the person with lived experience (eg, when an adult is presenting their story to adolescents [48]). We argue that a program that makes use of a video game, which is combined with a contact session with someone with lived experience, could address these limitations. In video games, adolescents can practice help-seeking and first aid behaviors in a safe but engaging environment [50]. Games are excellent learning tools as they provide immediate feedback on players’ actions, encouraging them to continue and learn more [51]. Games also allow for contact with people who youths normally might not often encounter, including peers with depression. Moreover, as games can be programmed to be played over a longer period, these interactions with peers with depression can be repeated over a period of multiple days. Games are also an important part of youths’ lives [52], making a game-based program relevant for young adolescents. In addition, there are already some successful examples of video games that teach adolescents health knowledge and skills [53,54] and decrease mental health stigma [55]. However, there are none that focus on both mental health literacy and stigma in youth. Finally, a contact session with someone with lived experience could add to the interactions that adolescents prefer, transfer the digital experience to the nondigital world, and further help decrease the stigma for depression. Especially when a program allows for informal discussions between adolescents and a person with lived experience, it could increase its effects on stigma [56]. For these reasons, we developed the game-based school program, *Moving Stories*, which combines mental health literacy training for depression with contact with someone with lived experience, both in the digital and nondigital world [20].

### This Study

The goal of this study is to conduct a first test of the effectiveness of the newly developed game-based program *Moving Stories* using a cluster randomized controlled trial [20]. Our primary hypotheses are that adolescents who participate in the *Moving Stories* program, compared with a control group (business as usual), will have better mental health literacy and lower stigma after the program and at the 3-month follow-up. Our secondary hypotheses concern the effectiveness of the *Moving Stories* program at the 6-month follow-up on mental health literacy and stigma and at the 3- and 6-month follow-ups on first aid and help-seeking behaviors. Specifically, we expect that adolescents who participate in the *Moving Stories* program will provide more appropriate first aid if they are in contact with a peer with a mental health problem and will be more likely to seek help personally if they themselves experience mental health symptoms compared with adolescents in the control group. Finally, we have included process measures to assess the use of the *Moving Stories* program and the possible side effects, including depressive symptoms.

## Methods

### Procedure

**Figure 1:**
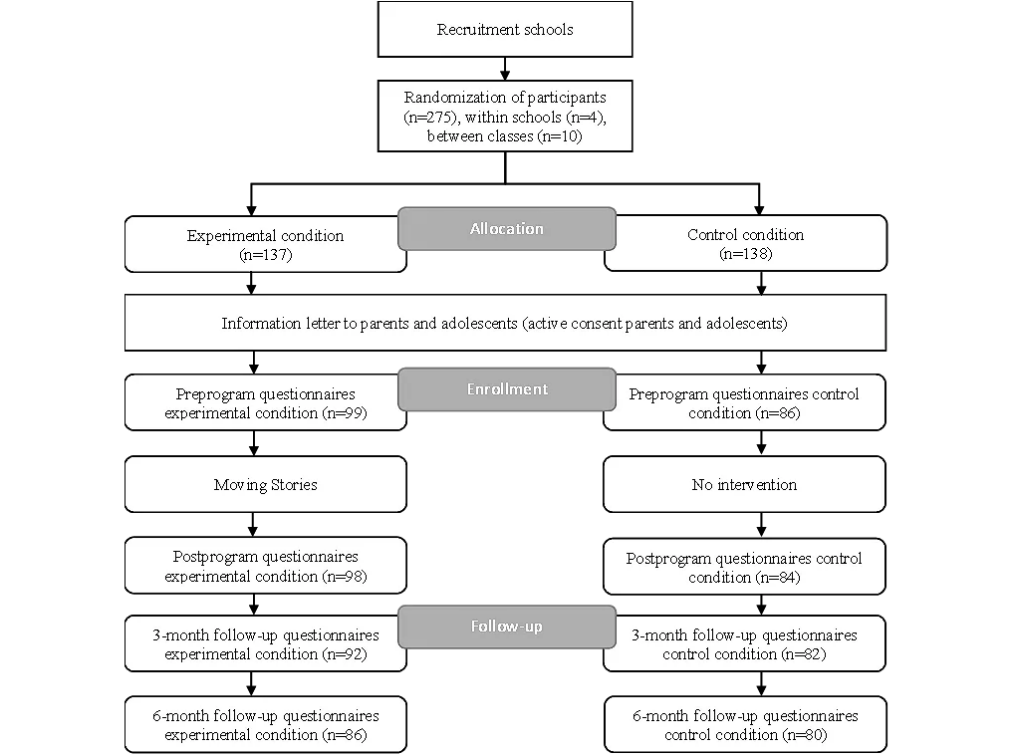
Flowchart of allocation, enrollment, and follow-up.

A total of 275 adolescents from 10 classes in 4 schools were asked to participate. Active informed consent was obtained; both adolescents and their parents received an information letter and consent form. The adolescents who did not get permission from their parents to participate or refused to participate themselves were excluded from the study and the program, leaving a sample of 185 participants (185/275, 67.3%). Parents and adolescents were allowed to withdraw their consent at any time. Figure 1 shows the flowchart of the allocation, enrollment, and follow-up of participants in the study. A summary of the procedure is presented in this section. For a more detailed explanation of the procedure, refer to the study protocol [20]. The CONSORT-EHEALTH (Consolidated Standards of Reporting Trials of Electronic and Mobile Health Applications and Online Telehealth) checklist (V 1.6.1) can be found in Multimedia Appendix 1.

The adolescents filled out digital questionnaires at 4 time points with a personal ID code and password (before the program, after the program, 3-month follow-up, and 6-month follow-up). Most adolescents completed the questionnaire at school. If they were not able to do so (eg, because of illness or because the school could not provide a time slot), they could complete the questionnaire at home. The adolescents who failed to fill in the questionnaire after the program or at the 3-month follow-up were allowed to participate in the subsequent time point. All dropouts were because of the adolescents forgetting or not willing to fill in the questionnaire at home, even after repeated reminders by phone or email. The adolescents received €12.50 (US $12.73) for completing at least the preprogram and 6-month follow-up questionnaires.

As *Moving Stories* was designed to motivate adolescents to talk about depression, the primary teachers of the participating classes and school care professionals (in both conditions) received an information booklet and information session in which possible actions were provided to discuss depressive symptoms or suicidal thoughts with students. They were given the opportunity to follow a web-based e-learning program on depression and suicidality in youth [57,58]. Furthermore, participants’ depressive symptoms were assessed at each time point to verify that *Moving Stories* did not increase depressive symptoms or suicidal ideation. If an adolescent had clinically high depressive symptoms or suicidal ideation, both the adolescent and their parents were contacted by phone by a clinically trained member of the research team to inform them of the result and give advice on where to seek professional help. In total, 4 adolescents and their parents were contacted. They did not withdraw from participating in the program or study.

### Ethics Approval

Ethical approval was provided by the ethics committee of the Faculty of Social Sciences at Radboud University (ECSW2017-2306-526). The study was registered in the Dutch Trial Register (NTR7033).

### Sample Size

We expected a small to medium effect (Cohen *d*=0.40) of *Moving Stories* on mental health literacy and stigma at the 3-month follow-up based on the results of a previous study with a school program for mental health literacy [43]. Our power analysis was based on our original analytic plan [20], using Stata (version 14.2; StataCorp) [59] and assuming baseline-adjusted regression analyses (*α*=.05; *β*=.20). Our provisional estimates for the correlations between pre- and postprogram measurements and between postprogram and 3-month follow-up measurements were 0.50. A coefficient of 0.19 (estimated mean cluster size 18; estimated cluster size range 11-25 [60]) and an intracluster correlation coefficient of 0.02 [43] led to a design effect of 1.35. Considering the design effect, we calculated that we needed 3.75 classes per condition to show the expected effect, rounding up to 4 classes per condition, with 18 adolescents per class. To adjust for a *t* distribution [61], we added 1 class per condition, resulting in 5 classes (ie, 90 adolescents) per condition and a total necessary sample size of 180.

### Program

Adolescents in the experimental condition participated in the *Moving Stories* program. *Moving Stories* is a game-based school program that comprises 3 parts: (1) an introduction lesson; (2) a single-player, mobile, and 3D video game [62]; and (3) a contact session with someone who has lived experience with a depressive disorder. For each class, the full program was delivered within 1 week. In the introduction lesson, adolescents were told what the video game was about (without using the term *depression*), and they were able to download and start the game with a classroom password. This password was linked to their class schedule to allow for joint playing time and shared feedback moments in the game.

**Figure 2:**
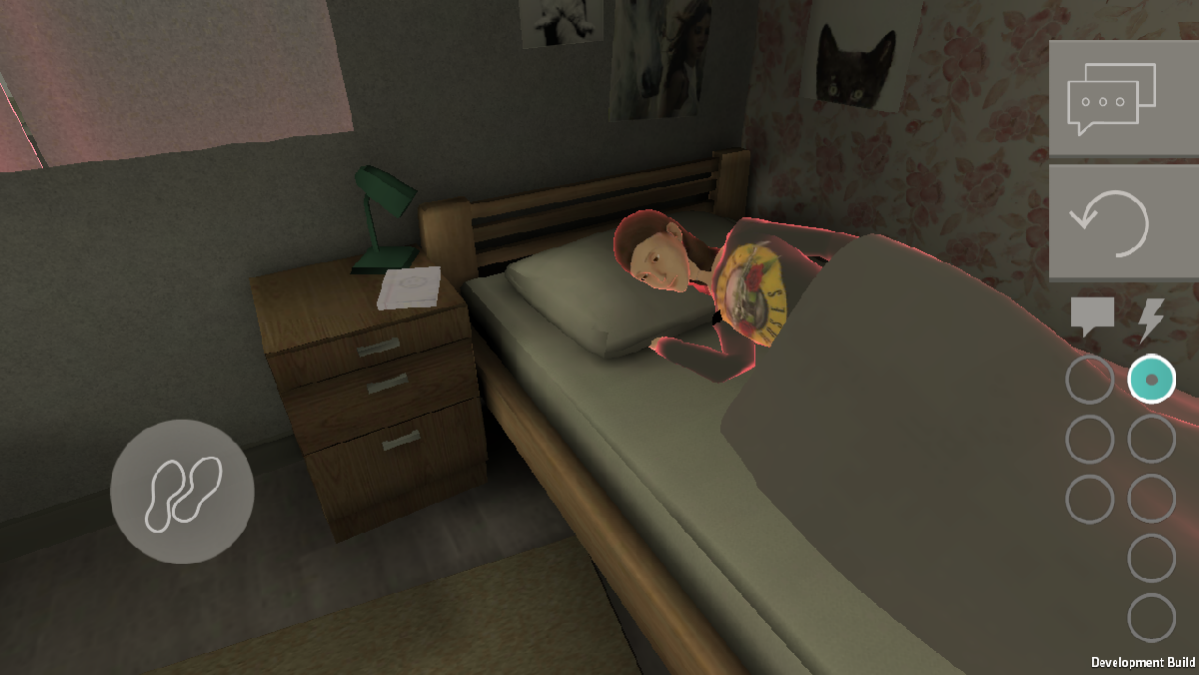
Screenshot of the *Moving Stories* video game.

The adolescents could play the game individually but in the same period as their classmates for 5 days in the morning (one of which was a weekend day). In the game, they interacted with the character Lisa (Figure 2), who showed signs of depression (without being labeled as depressive symptoms). Lisa’s symptoms were based on symptoms that are most common in adolescent girls in the Netherlands who have subclinical or clinical depression. These symptoms were derived from multiple data sets from Dutch studies on depression during adolescence. The adolescents were asked to help Lisa and were able to do so by performing 5 actions each day. Some of these actions had a positive effect on their relationship with Lisa, and others had a negative effect. During the day, at set time points, the adolescents received messages from Lisa with feedback on their actions. After 5 days of playing, the adolescents were able to see a final scene in which Lisa explained that she got help and thanked the player for their efforts in trying to help her.

The program ended with a contact session for the whole class, which was led by trainers with lived experience with depression. The trainers told their own life stories about depression. Using this life story and the experience the adolescents had in the game, the trainer discussed 5 first aid skills that the adolescents could use when one of their friends would experience depressive feelings. A detailed description of the program is available in the study protocol [20]. The adolescents in the control condition did not participate in any part of the *Moving Stories* program. They only filled in the questionnaires, and their teachers received information on depression and suicide as described in the *Procedure* section.

### Measures

#### Overview

We deviated from our study protocol by calculating McDonald *ω* instead of Cronbach *α* to assess the reliability of the measures. Many scholars have argued that in most psychological research, the assumptions made by Cronbach *α* are violated (eg, *τ* equivalence, normality, and a unidimensional scale), and that *ω* is a better alternative that can deal with these violations [63-66]. We used the package psych in R (R Foundation for Statistical Computing) to calculate the *ω* total [67]. We did not test the external validity of the various outcome measures. These concepts have been studied in multiple studies on mental health literacy and are related to behavioral outcomes. For example, in a study by Yap and Jorm [68], the researchers found that mental health first aid intentions predicted helping actions 2 years later.

#### Outcome Measures

##### Overview

Mental health literacy regarding depression was measured by (1) symptom recognition, (2) first aid intentions, (3) knowledge of first aid, (4) first aid confidence, (5) beliefs about help, and (6) help-seeking intentions. Depression stigma was measured by (1) personal stigma, (2) perceived stigma, and (3) social distance. A full description of all the measures is given in the study protocol [20].

##### Symptom Recognition

Symptom recognition was assessed using 3 vignettes with gender-matched descriptions of adolescents aged 15 years with depression, social anxiety, and psychosis [20,30]. Recognition of depression was defined by labeling the person in the depression vignette as depressed, whereas overestimation of depression was defined by labeling the person in the social anxiety or psychosis vignette as depressed. Responses to the vignettes were coded by undergraduate students (2 per vignette), and incongruencies were discussed. For all the following measures (unless otherwise mentioned), the vignette of the person with depression was used as an example.

##### First Aid Confidence

Confidence in providing first aid was measured by asking how confident the participant would be to help the person in the vignette if they were a friend [47].

##### First Aid Intentions and Skills

To measure general first aid intentions, participants were asked how much they agreed with the following statement: “If [name of the person in the vignette] was a friend, I would help him/her.” Specific first aid skills were assessed by asking whether the participant would perform each action on a list of 12 helpful and harmful first aid actions [44]. The scores for the harmful actions were reverse-scored. The helpful and harmful action scales have both shown acceptable to good reliability in a previous study [44]. However, in this sample, reliability was low for the 2 separate scales. We added up the helpful and reverse-scored harmful items to create a total score for first aid skills, with higher scores representing better first aid skills. The value of *ω* was between 0.71 and 0.82 for all 4 time points.

##### Beliefs About Help

Beliefs about help were assessed by asking whether a specific person would make the person’s situation in the vignette “better”; “not better”, “not worse”; or “worse.” A score for beliefs about appropriate help was calculated by adding up the number of selected adult sources (ie, parent, other relative, psychologist or social worker, phone helpline, general practitioner, teacher, school welfare coordinator or school counselor, and religious leader) deemed to be helpful (better) [47]. The value of *ω* was between 0.67 and 0.74 for all 4 time points.

##### Help-Seeking Intentions

Help-seeking intentions were measured using the General Help-Seeking Questionnaire, which has good internal consistency and excellent test–retest reliability [69]. Average scores for the following 3 scales were calculated: (1) general help-seeking intentions (all categories; *ω* between 0.78 and 0.82), (2) help-seeking intentions toward informal sources (categories 1-4; *ω* between 0.70 and 0.74), and (3) help-seeking intentions toward formal sources (categories 5-10; *ω* between 0.84 and 0.85). Higher scores indicated higher intentions.

##### Stigma

Stigma was measured with 3 scales. Both personal and perceived stigma was measured using the Dutch Depression Stigma Scale [70,71], whereas social distance was measured with the 5 items from the Social Distance Scale for youths [71]. The original personal and perceived stigma scales have both shown acceptable to good internal consistency [72], whereas the Social Distance Scale has shown excellent internal consistency [44]. In this sample, *ω* was between 0.62 and 0.67 for personal stigma, between 0.74 and 0.83 for perceived stigma, and between 0.84 and 0.87 for social distance. Higher scores for personal and perceived stigma indicated higher stigma, whereas higher scores for social distance indicated lower stigma.

##### First Aid Behavior

First aid behavior was measured by asking whether the participant has had contact with someone who has experienced a problem similar to the one seen in the vignette ever (before the program) or within the past 3 months (at 3- and 6-month follow-ups). If the participant answered *yes* or *maybe*, they were then asked whether they offered the other person their help. If so, or if they were unsure, they were asked what they did out of the 12 actions and the open-ended option mentioned in the *First Aid Intentions and Skills* section [44].

##### Help-Seeking Behavior

Help-seeking behavior was assessed by asking whether the participants themselves had experienced a problem similar to the situation in the vignette. If they responded with *yes* or *not sure*, they were asked whether someone had helped them with this problem in the past 3 months and who this person was. Multiple answers could be provided.

#### Distal Measure

Depressive symptoms were assessed with the Children’s Depression Inventory [73,74]. The Children’s Depression Inventory comprises 27 items, each with 3 statements to choose from. The participants were asked to pick the statement that best described how they felt in the previous 2 weeks.

#### Process Measures

##### Contamination Check

To check for possible contamination effects because of the within-school randomization, participants in the control group were asked at the 6-month follow-up whether they had heard of the game *Moving Stories* and, if so, whether they had played it. If they indicated that they had played it, they were asked on what platform they had done so.

##### Evaluation of Moving Stories

After the program, participants in the intervention group were asked to evaluate the program *Moving Stories* with 7 items. To distinguish between the different components of the program and the study (ie, game, evaluation session, and research), the participants were also asked which of the components they would recommend to a friend if they had the opportunity to participate in the study over the following year.

### Statistical Analyses

Data preparation and descriptive analyses were performed using SPSS Statistics (version 25; IBM Corp) [75]. All other analyses were conducted using R (version 3.5.1) [76]. We used multilevel analyses in R instead of regression analyses according to the intention-to-treat principle in Mplus [77], as described in the study protocol. Multilevel analyses in R were chosen for 2 reasons: (1) because our data were nested within participants and classes and therefore the analyses needed to account for this nested structure and (2) because multilevel analyses do not delete participants listwise, nor are imputations of data needed [78,79]. We ran linear mixed-effects models for all continuous outcome variables from preprogram measurements to postprogram measurements, 3-month follow-up, or 6-month follow-up and generalized linear mixed-effects models for all binary outcome variables using the R lme4 package [80]. We included random intercepts per participant and per class, as well as a random slope for condition per class. Therefore, our random effects structure modeled (1) baseline differences between participants, (2) baseline differences between classes, and (3) differences in the effectiveness of *Moving Stories* between classes. By including random effects, we knew that the differences between the experimental group and the control group would not be biased by differences between the classes. Therefore, the random effects structure benefited the interpretation in this 2-group design. When random effect variances were estimated as 0 in one of our models, we simplified the random effect structure of the model by first omitting random correlations and, if necessary, the random intercept for class. As recommended for confirmatory hypothesis testing, the random slope for our predictor of interest (ie, condition) was never omitted [81]. Time was set as a factor, and all categorical predictors were coded using sum-to-zero contrasts. All continuous predictors were standardized into *Z* scores. The significance of the coefficients was tested using *t* tests (2-tailed), with correction of the df based on the Satterthwaite approximation. A chi-square test was used to test if the condition had an effect on whether adolescents had provided help to someone in their network with a mental health problem. We reported the estimate, 95% CI, and 2-tailed *t* test results with *P*<.05 for the linear mixed-effects models and the odds ratio, 95% CI, and *Z* test results with *P*<.05 for the generalized linear mixed-effects models. The other results can be found in Multimedia Appendix 2 and the materials at the Open Science Framework [82].

### Open Science

All materials have been made publicly available via Open Science Framework [82]. The design and analysis plans for the experiments were published before finishing data collection at *JMIR Research Protocols* [20]. Deviations from the original analysis plans are described in this manuscript or in the materials at Open Science Framework.

## Results

### Participants

A total of 185 adolescents participated, with an age range of 12 to 15 years (mean 13.43, SD 0.67 years). Of the 185 participants, 101 (54.6%) were boys, and 179 (96.8%) were born in the Netherlands. The educational levels of the classes were either only preparatory secondary vocational education or preparatory secondary vocational education including higher general secondary education (low to middle level; 72/185, 38.9%) and preuniversity education (highest level; 113/185, 61.1%). Approximately 91.9% (170/185) of the adolescents indicated that they played video games and did so for an average of 8.48 (SD 7.26) hours per week.

Dropout at postprogram measurements (3/185, 1.6%) and the 3-month follow-up was low (11/185, 5.9%). At the 6-month follow-up, approximately 89.7% (166/185) of the adolescents finished the questionnaire (10% dropout). There were no significant differences in study condition, gender, country of birth, and gaming behavior and frequency among adolescents who dropped out at the 6-month follow-up and adolescents who finished the final questionnaire. However, the adolescents who did not finish the final questionnaire were significantly older (t_183_=2.47; *P*=.01) and had higher depressive symptoms (t_183_=2.63; *P*=.009) and a lower educational level (*χ*^2^_1_=18.3; *P*<.001) than the adolescents who finished the final questionnaire.

### Baseline Differences

Differences were found between the experimental and control groups in terms of gender (*χ*^2^_1_=10.5; *P*=.001), whether the adolescents generally played video games (*χ*^2^_1_=7.4; *P*=.007), and number of hours playing video games (t_181.98_=2.93; *P*=.004). In the experimental group, 66% (65/99) of the participants were male, whereas in the control group, 42% (36/86) were male. Significantly more adolescents in the experimental group indicated that they played video games, and they did so more hours a week than those in the control group. We included gaming frequency (standardized) and gender as covariates in all models. Tables 1 and 2 show the descriptives of the outcomes for both conditions. At preprogram measurements, the experimental group had less adequate first aid skills than the control group (t_182.35_=−2.09; *P*=.04) and more social distance toward peers with depression (t_183_=−2.04; *P*=.04). Moreover, the experimental group deemed more adults to be helpful than the control group (t_183_=2.07; *P*=.04).

**Table 1:** Percentages of adolescents who mentioned depression at the 3 vignettes (N=185).

Depression mentioned in vignette per group		Preprogram measurement		Postprogram measurement		3-month follow-up			6-month follow-up	
		n (%)	N	n (%)	N	n (%)	N	n (%)		N
**Depression vignette**										
	Moving Stories	62 (63)	99	90 (92)^a^	98	70 (76)^a^	92	64 (74)		86
	Control	57 (66)	86	75 (89)^a^	84	71 (87)^a^	82	68 (85)		80
**Social anxiety vignette**										
	Moving Stories	0 (0)	99	2 (2)^a^	98	1 (1)^a^	92	1 (1)		86
	Control	1 (1)	86	1 (1)^a^	84	0 (0)^a^	82	1 (1)		80
**Psychosis vignette**										
	Moving Stories	13 (13)	99	34 (35)^a^	98	29 (32)^a^	92	21 (24)		86
	Control	9 (10)	86	13 (15)^a^	84	17 (21)^a^	82	20 (25)		80

^a^Primary outcomes.

**Table 2:** Means and SDs of the outcomes of the continuous variables.

Outcome and group			Preprogram measurement, mean (SD)		Postprogram measurement, mean (SD)		3-month follow-up, mean (SD)		6-month follow-up, mean (SD)
**First aid confidence**									
	Moving Stories	3.60 (0.89)		3.48 (1.02)^a^		3.47 (1.03)^a^		3.29 (1.10)	
	Control	3.48 (1.03)		3.49 (1.02)^a^		3.46 (1.08)^a^		3.55 (1.01)	
**General first aid intentions**									
	Moving Stories	4.45 (1.02)		4.27 (0.97)^a^		4.25 (0.88)^a^		4.22 (1.01)	
	Control	4.42 (0.93)		4.25 (1.02)^a^		4.23 (1.01)^a^		4.40 (0.92)	
**Specific first aid skills**									
	Moving Stories	43.12 (4.61)		43.00 (4.76)^a^		42.55 (4.74)^a^		42.57 (4.47)	
	Control	44.41 (3.77)		43.18 (4.87)^a^		43.87 (4.63)^a^		43.59 (4.38)	
**Beliefs about help**									
	Moving Stories	3.03 (1.63)		2.97 (1.79)^a^		2.45 (1.63)^a^		2.51 (1.70)	
	Control	2.56 (1.45)		2.36 (1.56)^a^		2.29 (1.51)^a^		2.39 (1.56)	
**Help-seeking intentions (total)**									
	Moving Stories	4.06 (0.98)		4.18 (1.02)^a^		3.98 (0.91)^a^		3.94 (0.83)	
	Control	3.87 (0.90)		3.86 (0.81)^a^		3.85 (0.75)^a^		3.85 (0.75)	
**Help-seeking intentions (informal)**									
	Moving Stories	4.83 (1.15)		4.86 (1.22)^a^		4.85 (1.07)^a^		4.85 (1.07)	
	Control	4.79 (1.14)		4.67 (1.08)^a^		4.61 (1.20)^a^		4.72 (1.08)	
**Help-seeking intentions (formal)**									
	Moving Stories	3.21 (1.23)		3.42 (1.34)^a^		3.08 (1.28)^a^		2.99 (1.23)	
	Control	2.93 (1.06)		3.00 (1.00)^a^		2.89 (0.94)^a^		2.93 (1.13)	
**Personal stigma**									
	Moving Stories	18.56 (3.83)		17.74 (3.36)^a^		17.14 (4.10)^a^		16.81 (4.06)	
	Control	17.57 (3.70)		17.87 (3.58)^a^		17.59 (3.55)^a^		16.79 (3.47)	
**Perceived stigma**									
	Moving Stories	22.71 (4.14)		22.24 (4.81)^a^		22.73 (4.90)^a^		22.62 (4.84)	
	Control	21.97 (4.48)		22.61 (4.70)^a^		22.01 (4.86)^a^		22.08 (4.28)	
**Social distance (stigma)**									
	Moving Stories	14.27 (2.78)		14.56 (2.82)^a^		13.87 (3.03)^a^		14.19 (2.97)	
	Control	15.13 (2.91)		14.54 (2.87)^a^		14.88 (2.99)^a^		14.58 (3.06)	

^a^Primary outcomes.

### Outcome Measures

#### Symptom Recognition

No effects of condition on recognition of depressive symptoms in the depression vignette or on overestimation of depressive symptoms in the psychosis vignette were found from preprogram to postprogram measurements and 3- and 6-month follow-ups. For the social anxiety vignette, variance was close to 0 (approximately no adolescents indicated they thought the vignette was about depression); therefore, we did not conduct any analyses on the social anxiety vignette.

#### First Aid Confidence

No effects of condition were found at the postprogram measurement and 3-month follow-up for first aid confidence. Participation in the experimental group predicted changes over time in first aid confidence from the preprogram measurement to the 6-month follow-up compared with the control group (*b*=−0.20, 95% CI −0.35 to −0.05; t_169.28_=−2.61; *P*=.01). Contrary to our secondary hypothesis, the experimental group had a decrease in first aid confidence from the preprogram measurement to the 6-month follow-up (t_86_=3.74; *P*<.001), whereas this effect was not found in the control group.

#### First Aid Intentions

Participation in the experimental group did not significantly predict changes over time in intentions for general or specific first aid skills.

#### Beliefs About Help

Participation in the experimental group did not significantly predict changes over time in beliefs about the helpfulness of adults.

#### Help-Seeking Intentions

Participation in the experimental group did not significantly predict changes over time in help-seeking intentions toward informal, formal, or all help sources.

#### Stigma

Participation in the experimental group, compared with the control group, was related to changes over time in personal stigma from pre- to postprogram measurements (*b*=−0.53, 95% CI −1.02 to −0.03; t_179.16_=−2.08; *P*=.04) and from preprogram measurements to the 3-month follow-up (*b*=−0.57, 95% CI −1.11 to −0.03; t_174.39_=−2.07; *P*=.04). At the postprogram measurements and 3-month follow-up, in line with our primary hypotheses, personal stigma decreased significantly in the experimental group (postprogram measurement: t_97_=2.40, *P*=.02; 3-month follow-up: t_91_=3.81, *P*<.001), whereas this was not the case for the control group. No effects of condition on perceived stigma and social distance were found at postprogram measurement and the 3- and 6-month follow-ups, and the effects on personal stigma did not last at the 6-month follow-up.

#### First Aid Behavior

There were no significant differences at the 3- and 6-month follow-ups between the experimental and control groups on whether adolescents had provided help to someone in their network with a mental health problem. At preprogram measurements, of the 71 participants who were in contact with someone in their network with a mental health problem, 66 (93%) indicated having offered help. At the 3-month follow-up, this was 89% (31/35 participants), and at the 6-month follow-up, it was 67% (24/36 participants).

#### Help-Seeking Behavior

There were no significant differences at the 3- and 6-month follow-ups between the experimental and control conditions on whether adolescents had a personal experience with a mental health problem. At preprogram measurements, 17.3% (32/185) of the participants indicated whether they ever had a mental health problem (or did not know for sure). At the 3-month follow-up, 10.9% (19/174) of the participants indicated having had a mental health problem in the previous 3 months (or did not know for sure), whereas at the 6-month follow-up, this was 5.4% (9/166) of the participants. None of the adolescents at the 3- and 6-month follow-ups mentioned that they had not been helped.

### Distal Measure

Participation in the experimental group was not related to changes in depressive symptoms over time.

### Process Measures

#### Contamination Check

In the control group, at the 6-month follow-up, 3% (2/80) of the adolescents indicated that they had heard of *Moving Stories*. None of them indicated they had played the game.

#### User Statistics

Most of the adolescents in the experimental group participated in the introduction and contact sessions (97/99, 98% and 93/99, 94% of participants, respectively). During the playing days, 49% (49/99) of the adolescents played the game for 5 days, 34% (34/99) played it for 4 days, 14% (14/99) played it for 3 days, and 3% (3/99) played it for ≤2 days.

#### Evaluation of Moving Stories

The adolescents in the experimental group rated *Moving Stories* positively at the postprogram measurements. When asked whether they would recommend elements of the study and program to a friend, 92% (90/98) of the adolescents indicated they would recommend the game, 66% (65/98) indicated they would recommend the contact session, and 79% (77/98) indicated they would recommend the research element. Of the 98 adolescents, 75 (77%) understood the game, 67 (68%) thought they had learned something from it, and 74 (76%) were interested in how Lisa would be doing in 6 months. Fewer adolescents rated the contact session positively (44/98, 45%); however, 53% (52/98) of the adolescents indicated that they had learned something from it. Approximately 40% (39/98) indicated that they would play the game a second time to get a better score. In terms of stigmatizing attitudes toward Lisa, 23% (23/98) of the adolescents indicated that they would not hang out with Lisa in daily life, whereas the others were neutral or positive toward her.

## Discussion

### Principal Findings

The goal of this cluster randomized controlled trial was to test the effects of the newly developed game-based school program *Moving Stories* [20] on mental health literacy and stigma regarding depression in adolescents.

We found that participation in the *Moving Stories* program reduced personal stigma over time compared with participation in the control condition. These results are in line with previous research showing that contact with someone with lived experience can reduce stigma [39]. Interacting with a video game character with depressive symptoms combined with meeting someone with lived experience in the *Moving Stories* program seemed to be similarly effective, at least in terms of immediate effects. However, improvements in personal stigma did not continue beyond 3 months, and the effects could be a result of regression to the mean [83]. These results suggest that repeated conversations about mental health in the classroom or increased interactions with people with mental health disorders (either virtually or face-to-face) may be needed to achieve longer-lasting improvements. Furthermore, implementing booster sessions with the game may be beneficial as well [84-86].

Contrary to our secondary hypothesis, we found that adolescents in the *Moving Stories* group were less confident about providing help at the 6-month follow-up compared with adolescents in the control group; however, there were no group differences at the 3-month follow-up or after the program. The adolescents in the *Moving Stories* group may have felt less confident after 6 months about remembering and applying the helpful skills taught in the *Moving Stories* program, whereas the adolescents in the control group had no knowledge of whether specific skills were helpful or harmful. Indeed, we did not find any effects on first aid skills. It could be that, for confidence to increase, a strong and longer-lasting knowledge improvement would be needed.

No group differences were found for symptom recognition, first aid intentions, beliefs about the helpfulness of adults in general, help-seeking intentions in general, or toward informal and formal sources specifically. We did not find group differences in perceived stigma, social distance, or providing and seeking help. The null results in our study are not in line with previous work, as other mental health literacy programs have been found to be effective in improving mental health literacy, specifically in terms of knowledge [43,44,47]. An explanation could be that our study was conducted on a population of younger adolescents. Most successful mental health literacy programs have been tested on older adolescents [43,44], and a study with younger adolescents found weaker effects [45]. Younger adolescents may be less developmentally equipped or lack experience with depressed peers to relate to the topic [45]. Another reason for not finding significant effects could be that the content of the *Moving Stories* program was not sufficient to improve mental health literacy. Other mental health literacy programs have incorporated didactic sessions in which facts about depression and first aid skills are explained. The *Moving Stories* program did not include didactic sessions but was focused on learning by doing [50]. It is possible that the experiences in the video game did not sufficiently lead to improved knowledge. As the contact session took approximately 50 minutes and focused on sharing experiences and translating the story of Lisa to the real world, providing information about depression was not prioritized. Future iterations of *Moving Stories* could combine the learning by doing in the video game with the didactic sessions from other programs to improve its effectiveness. This might be especially beneficial for a younger, less-experienced group of adolescents. Finally, it could also be that we did not have sufficient power to show effects in a Dutch context. We based our power analysis on an Australian study; however, the contexts of the 2 countries might have differed too much. Future studies in a Dutch context with *Moving Stories*, or any other mental health literacy program, should start with a lower expected effect size.

Finally, the process evaluation showed that *Moving Stories* was well-received by the adolescents, and adherence to the full program was high. This is as important as the stigma results, as both digital and nondigital mental health programs often suffer from engagement problems [87]. If we want a school program to be successful outside the research context, we need adolescents to be intrinsically motivated to participate in the program. Although the *Moving Stories* program does not have the same entertainment goals as the video games adolescents play today, adolescents in our study were still engaged by the game and interested in the content of both the game and the contact session.

### Strengths and Limitations

This study used a rigorous design to test the effects of a new mental health literacy program. Most studies on the effects of health literacy programs do not include a control group, and very few studies test the effects over a longer period [40-42]. Another strength of this study is the assessment of ≥1 element of mental health literacy [21]. Many studies on mental health literacy focus solely on knowledge of depression facts [88]. In this study, we also assessed knowledge of available help, first aid skills, and help-seeking and first aid intentions.

A limitation of our study was the difference between dropouts and completers at the 6-month follow-up. Age and education level differences in attrition were mostly because of a class that received the questionnaire right before the Christmas break and therefore received fewer reminders. Compared with the other classes, adolescents in this class had a higher mean age and lower educational level. However, this particular class did not explain the attrition differences found in depressive symptoms. Adolescents with depressive symptoms may have been less comfortable with answering questions about their mental state and therefore did not fill in subsequent questionnaires [89-91]. In addition, all participants were made aware that in the exceptional case that the data showed that the adolescent was *feeling very bad*, they themselves and their parents would be contacted by the research team. To avoid such a call, the adolescents could have chosen not to fill out later questionnaires.

A second limitation was the relatively low reliability of some of the outcome measures (ie, beliefs about help and personal stigma). These outcomes should be interpreted with caution. In general, low psychometric quality is found for mental health literacy measures [88] and, to enable comparisons, we included questionnaires that were used in studies on one of the most rigorously evaluated mental health literacy programs (*teen Mental Health First Aid* [44]). Although the use of vignettes had the benefit of not relying on labels to assess mental health first aid, a disadvantage was that we did not know what the effects of repeated use of the vignettes were. However, our study did show a variety in responses to the vignettes over time, meaning that the participants did not simply repeat their answers. More work is needed to develop measures of good quality that help us assess the effects of mental health literacy programs more effectively [88].

Finally, we were unable to explore the behavioral effects of the *Moving Stories* program because of small subsamples and differences at baseline in the quality of the provided help between the 2 conditions. If we are interested in studying whether adolescents show appropriate and effective behavioral responses when coming into contact with a friend with depressive symptoms after participation in a mental health literacy program, long-term follow-ups with larger samples are needed to allow for the occurrence of such an event. Moreover, assessing reaching out for help next to assessing receiving help would provide even more useful information.

### Future Directions

On the basis of the results of this study, we have several recommendations for future directions of the *Moving Stories* program specifically and mental health literacy programs in general. One recommendation is to compare the effects of a mental health literacy program between younger and older adolescents. The original idea of *Moving Stories* was to teach adolescents first aid skills and help-seeking behavior before most mental health problems occurred. However, it could be that a mental health literacy program is more effective when students have experienced mental health problems themselves or in their social environment, which is more likely when they are older. Another recommendation is to tailor mental health programs to the preferences of the users. This becomes a more feasible option when digital tools are used and might improve their effectiveness [87]. For example, for *Moving Stories*, we could create a male or nonbinary version of Lisa and increase the number of first aid and dialog options in the game. Then, the game could be tailored to the preferences of a user but also to their knowledge and experience level. Finally, although many programs, both for mental health literacy and stigma, include the presence of a person with lived experience, the organization of these sessions on a large scale has proven to be difficult. Other options could be to include video material of people with lived experience while a school mental health professional leads a discussion. An added benefit is that the students participating in these discussion sessions are immediately in contact with someone who can provide help if they need it.

### Conclusions

The results of this clustered randomized controlled trial seem to show that the *Moving Stories* program could be effective in reducing personal stigma regarding depression in adolescents, although the effects were small. Moreover, the program was well-received and well-adhered to, something that can be difficult to achieve with young adolescents. In its current form, the *Moving Stories* program had no effect on mental health literacy components, perceived stigma, or social distance. Our results suggest that a larger sample might be needed to show the effects on a Dutch adolescent population. Nevertheless, the stigma results and positive evaluations may be a good stepping stone for further iterations of the program. Combining the program with didactic elements from other programs and scheduling regular follow-up conversations in the classroom might improve these results. Moreover, tailoring the video game to each individual player’s preferences and skill level could further increase its effects. With better results and after showing its effectiveness in studies with larger sample sizes, the program could be implemented in schools to improve help-seeking in adolescents and reduce the negative consequences and burden of depressive symptoms.

## Appendix

Multimedia Appendix 1 CONSORT-EHEALTH (Consolidated Standards of Reporting Trials of Electronic and Mobile Health Applications and Online Telehealth) checklist (V 1.6.1).

Multimedia Appendix 2 Test results linear mixed effects models.

## Abbreviations

CONSORT-EHEALTH: Consolidated Standards of Reporting Trials of Electronic and Mobile Health Applications and Online Telehealth
