# Supplementary material for: A Game-Based School Program for Mental Health Literacy and Stigma on Depression (Moving Stories): Cluster Randomized Controlled Trial
Source: JMIR Ment Health. 2022 Aug 17;9(8):e26615. doi: 10.2196/26615 (PMC9434393; doi:10.2196/26615)
Supplement: Multimedia Appendix 2 [file mental_v9i8e26615_app2.docx]

**Multimedia Appendix 2 Test results linear mixed effects models.**

**Table 1.** Odds ratio (OR), 95% confidence interval (CI), z-test result and p-value for the generalized linear mixed effects models.

|  | | OR | 95% CI | z | P |
| --- | --- | --- | --- | --- | --- |
| Recognition of depressive symptoms | | | | | |
|  | From pre- to postprogram measurement ^a^ | 1.59 | [0.95 – 2.73] | 1.73 | .08 |
|  | From preprogram measurement to 3-month follow-up ^a^ | 0.90 | [0.57 – 1.42] | -0.47 | .64 |
|  | From preprogram measurement to 6-month follow-up | 0.83 | [0.53 – 1.30] | -0.81 | .42 |
| Recognition of psychosis symptoms | | | | | |
|  | From pre- to postprogram measurement ^a^ | 1.94 | [0.96 – 4.10] | 1.83 | .07 |
|  | From preprogram measurement to 3-month follow-up ^a^ | 1.37 | [0.70 – 2.73] | 0.92 | .36 |
|  | From preprogram measurement to 6-month follow-up | 0.97 | [0.52 – 1.79] | -0.08 | .93 |

^a^Primary outcomes.

**Table 2a.** Estimate, 95% confidence interval (CI), t-test result and p-value for the linear mixed effects models.

|  | | b | 95% CI | t | df | P |
| --- | --- | --- | --- | --- | --- | --- |
| First aid confidence | | | | | | |
|  | From pre- to postprogrammeasurement ^a^ | -0.05 | [-0.26 – 0.15] | -0.51 | 349.31 | .61 |
|  | From preprogram measurement to 3-month follow-up ^a^ | -0.07 | [-0.28 – 0.15] | -0.60 | 345.75 | .55 |
|  | From preprogram measurement to 6-month follow-up | -0.20 | [-0.35 – -0.05] | -2.61 | 169.28 | .01 |
| General first aid intentions | | | | | | |
|  | From pre- to postprogrammeasurement ^a^ | 0.01 | [-0.20 – 0.21] | 0.06 | 359.00 | .95 |
|  | From preprogram measurement to 3-month follow-up ^a^ | 0.01 | [-0.19 – 0.22] | 0.13 | 352.00 | .90 |
|  | From preprogram measurement to 6-month follow-up | -0.09 | [-0.27 – 0.10] | -0.90 | 173.62 | .37 |

^a^Primary outcomes.

**Table 2b.** Estimate, 95% confidence interval (CI), t-test result and p-value for the linear mixed effects models.

|  | | b | 95% CI | t | df | P |
| --- | --- | --- | --- | --- | --- | --- |
| Specific first aid skills | | | | | | |
|  | From pre- to postprogrammeasurement ^a^ | 0.56 | [0.00 – 1.12] | 1.95 | 178.26 | .05 |
|  | From preprogram measurement to 3-month follow-up ^a^ | 0.14 | [-0.76 – 1.05] | 0.30 | 347.55 | .76 |
|  | From preprogram measurement to 6-month follow-up | 0.04 | [-0.86 – 0.94] | 0.09 | 344.00 | .93 |
| Beliefs about help | | | | | | |
|  | From pre- to postprogrammeasurement ^a^ | 0.10 | [-0.12 – 0.32] | 0.86 | 179.62 | .39 |
|  | From preprogram measurement to 3-month follow-up ^a^ | -0.13 | [-0.37 – 0.10] | -1.10 | 177.70 | .27 |
|  | From preprogram measurement to 6-month follow-up | -0.19 | [-0.44 – 0.07] | -1.42 | 169.47 | .16 |
| Help-seeking intentions, total | | | | | | |
|  | From pre- to postprogrammeasurement ^a^ | 0.06 | [-0.14 – 0.25] | 0.58 | 355.64 | .56 |
|  | From preprogram measurement to 3-month follow-up ^a^ | <.01 | [-0.13 – 0.13] | <-0.01 | 177.30 | 1.00 |
|  | From preprogram measurement to 6-month follow-up | -0.06 | [-0.25 – 0.12] | -0.68 | 339.51 | .49 |
| Help-seeking intentions, informal | | | | | | |
|  | From pre- to postprogrammeasurement ^a^ | 0.07 | [-0.17 – 0.30] | 0.54 | 354.94 | .59 |
|  | From preprogram measurement to 3-month follow-up ^a^ | 0.05 | [-0.13 – 0.24] | 0.57 | 179.83 | .57 |
|  | From preprogram measurement to 6-month follow-up | 0.01 | [-0.22 – 0.25] | 0.11 | 338.55 | .91 |
| Help-seeking intentions, formal | | | | | | |
|  | From pre- to postprogrammeasurement ^a^ | 0.06 | [-0.19 – 0.30] | 0.46 | 356.70 | .65 |
|  | From preprogram measurement to 3-month follow-up ^a^ | -0.04 | [-0.29 – 0.20] | -0.34 | 351.00 | .74 |
|  | From preprogram measurement to 6-month follow-up | -0.11 | [-0.36 – 0.13] | -0.90 | 340.58 | .37 |

^a^Primary outcomes.

**Table 2c.** Estimate, 95% confidence interval (CI), t-test result and p-value for the linear mixed effects models.

|  | | b | 95% CI | t | df | P |
| --- | --- | --- | --- | --- | --- | --- |
| Personal stigma | | | | | | |
|  | From pre- to postprogrammeasurement ^a^ | -0.53 | [-1.02 – -0.03] | -2.08 | 179.16 | .04 |
|  | From preprogram measurement to 3-month follow-up ^a^ | -0.57 | [-1.11 – -0.03] | -2.07 | 174.39 | .04 |
|  | From preprogram measurement to 6-month follow-up | -0.49 | [-1.01 – 0.03] | -1.83 | 169.35 | .07 |
| Perceived stigma | | | | | | |
|  | From pre- to postprogrammeasurement ^a^ | -0.61 | [-1.31 – 0.09] | -1.70 | 179.75 | .09 |
|  | From preprogram measurement to 3-month follow-up ^a^ | 0.03 | [-0.78 – 0.84] | 0.08 | 178.95 | .94 |
|  | From preprogram measurement to 6-month follow-up | -0.26 | [-1.05 – 0.54] | -0.63 | 177.24 | .53 |
| Social distance (stigma) | | | | | | |
|  | From pre- to postprogrammeasurement ^a^ | 0.32 | [-0.25 – 0.89] | 1.10 | 350.26 | .27 |
|  | From preprogram measurement to 3-month follow-up ^a^ | -.17 | [-0.76 – 0.44] | -0.53 | 344.33 | .59 |
|  | From preprogram measurement to 6-month follow-up | 0.12 | [-026 – 0.51] | 0.63 | 170.40 | .53 |

^a^Primary outcomes.
